# Ex Vivo Evaluation of Glutamine Treatment in Sepsis and Trauma in a Human Peripheral Blood Mononuclear Cells Model

**DOI:** 10.3390/nu15010252

**Published:** 2023-01-03

**Authors:** Efrossini Briassouli, Marianna Tzanoudaki, Dimitris Goukos, Kostas Vardas, Panagiotis Briassoulis, Stavroula Ilia, Maria Kanariou, Christina Routsi, Serafim Nanas, George L. Daikos, George Briassoulis

**Affiliations:** 1 Department of Immunology-Histocompatibility, Specialized Center & Referral Center for Primary Immunodeficiencies-Paediatric Immunology, “Aghia Sophia” Children’s Hospital, 11527 Athens, Greece; 2 First Department of Propaedeutic Medicine, School of Medicine, National and Kapodistrian University of Athens (NKUA), 11527 Athens, Greece; 3 Infectious Diseases Department “MAKKA”, “Aghia Sophia” Children’s Hospital, First Department of Paediatrics, School of Medicine, National and Kapodistrian University of Athens (NKUA), 11527 Athens, Greece; 4 First Critical Care Department, Evangelismos Hospital, School of Medicine, National and Kapodistrian University of Athens (NKUA), 15772 Athens, Greece; 5 Attikon University Hospital, School of Medicine, National and Kapodistrian University of Athens (NKUA), 12462 Athens, Greece; 6 Pediatric Intensive Care Unit, University Hospital, School of Medicine, University of Crete, 71110 Heraklion, Greece; 7 Postgraduate Program “Emergency and Intensive Care in Children Adolescents and Young Adults”, School of Medicine, University of Crete, 71003 Heraklion, Greece

**Keywords:** glutamine, heat shock, lipopolysaccharide, heat shock protein, mRNA, flow cytometry, peripheral blood mononuclear cells, cytokines, interleukin, MCP-1, sepsis, trauma, critically ill

## Abstract

We aimed to assess the lipopolysaccharide (LPS), or heat shock (HS) induction, and glutamine-modulating effects on heat shock protein-90α (HSP90α) and cytokines in an ex vivo model using peripheral blood mononuclear cells (PBMCs). The PBMCs of patients with septic shock, trauma-related systemic inflammatory response syndrome (SIRS), and healthy subjects were incubated with 1 μg/mL LPS at 43 °C (HS). Glutamine 10 mM was added 1 hour before or after induction or not at all. We measured mRNA HSP90α, monocyte (m) and lymphocyte (l) HSP90α proteins, interleukin (IL)-1b, -6, -8, -10, tumor necrosis factor-α (TNF-α), and monocyte chemoattractant protein-1 (MCP-1) supernatant levels. Heat shock increased the HSP90α mRNA and mHSP90α in all groups (10-fold in sepsis, *p* < 0.001 and *p* = 0.047, respectively). LPS induced the mHSP90α and lHSP90α in healthy (*p* < 0.001) and mHSP90α in SIRS (*p* = 0.004) but not in sepsis. LPS induced the cytokines at 24 and 48 h in all groups, especially in trauma (*p* < 0.001); HS only induced the IL-8 in healthy (*p* = 0.003) and septic subjects (*p* = 0.05). Glutamine at 10 mM before or after stimulation did not alter any induction effect of LPS or HS on HSP90α mRNA and mHSP90α protein in sepsis. In SIRS, glutamine before LPS decreased the mHSP90α but increased it when given after HS (*p* = 0.018). Before or after LPS (*p* = 0.049) and before HS (*p* = 0.018), glutamine decreased the lHSP90α expression in sepsis but increased it in SIRS when given after HS (*p* = 0.003). Regarding cytokines, glutamine enhanced the LPS-induced MCP-1 at 48 h in healthy (*p* = 0.011), SIRS (*p* < 0.001), and sepsis (*p* = 0.006). In conclusion, glutamine at 10 mM, before or after LPS and HS, modulates mHSP90α and lHSP90α in sepsis and SIRS differently and unpredictably. Although it does not alter the stimulation effect on interleukins, glutamine enhances the LPS induction effect on supernatant MCP-1 in all groups. Future research should seek to elucidate better the impact of glutamine and temperature modulation on HSP90α and MCP-1 pathways in sepsis and trauma.

## 1. Introduction

Sepsis is defined as infection-related organ dysfunction resulting from host response dysregulation [1]. Multiple organ system failures and increased mortality rates are associated with septic shock [2]. In sepsis and systemic inflammatory response syndrome (SIRS), a rapid-onset cytokine storm leads to a rapid release of pro-inflammatory mediators and an excessive release of anti-inflammatory cytokines [3]. Trauma-related SIRS represents another leading cause of morbidity and mortality. 

Immune cells such as lymphocytes and macrophages release pro-inflammatory biomolecules such as tumor necrosis factor-α (TNF-α), interleukin (IL)-1, IL-6, and IL-8 followed by the production of anti-inflammatory mediators such as IL-10, IL-13, or transforming growth factor-β (TGF- β) [3]. By responding to the cytokine-storm, core temperature rises above a threshold set by the hypothalamus, causing cellular damage and death [4]. The clinically evident hyperthermia (fever) involves heat shock (HS) pathways, including the HS proteins (HSP) 70 and 90. Although large clinical trials on targeted temperature management have not shown benefits in sepsis, national guidelines recommend normothermia in patients with trauma [5]. In a large adult cohort, sepsis-related fever was associated with worse outcome [6], whereas fever control using external cooling to maintain normothermia reduced mortality in septic shock [3]. Surprisingly, external forced-air warming of normothermic septic patients was associated with lower mortality when compared to afebrile patients [7]. 

The highly conserved defence HSPs protect cells from injury, repair cell damage, and enhance cell tolerance to stress by rapidly responding to various insults [8]. A major molecular chaperone, the 90-kDa family of HSP, functions as an ATP-dependent molecular chaperone guiding the late-stage tertiary folding of more than 200 client proteins [9]. In humans, the extracellular HSP90α is dramatically increased in septic shock, related to HSP72, nCD64, cytokines, and hyperthermia [9,10,11]. 

Immune-modulating nutrients have been shown to affect the expression of HSPs, serving as extracellular chaperokines and intracellular chaperones [12]. Although animal studies showed a strong protective effect of HSP70 in sepsis, human studies are inconclusive [13]. Glutamine, representing 20% of the total free amino acids pool in the blood [14], is a pharmaco-nutrient modulating inflammatory and immune stress responses [15]. Under acute stress conditions, such as sepsis or SIRS, glutamine is utilized as a vital fuel for the immune system at similar rates to glucose utilization [16]. Endogenous glutamine synthesis, however, cannot meet the increased demands in catabolic conditions, such as sepsis [17,18] and trauma [19]. 

Glutamine supplementation of enteral nutrition induced HSP90α-attenuated lymphocyte apoptosis and enhanced the immunological function in severely burned rats [20]. Sepsis-induced rat cardiac myocyte apoptosis was reduced by glutamine supplementation and overexpression of HSP90α [21]. Despite the encouraging experimental data, large-scale trials have suggested harm associated with glutamine treatment. The REDOXS [22] and the MetaPlus [23] trials showed increased mortality in intensive care unit (ICU) patients supplemented with glutamine. Additionally, a systematic review showed that glutamine does not benefit ICU patients except for reduced hospital stay [24]. Randomized studies, however, suggested that glutamine decreases gastric colonization, infectious complications, and ICU length of stay [25,26,27,28]. Further confusing its role, elevations in glutamine along with proline, glycine, methionine, and two acylcarnitines were not only related to organ-support-free days but also chronic critical illness development [29]. 

Smart-trials are needed to more accurately define possible interrelations of glutamine with acute inflammatory and innate immune responses and identify specific sub-cohorts of critically ill patients who might benefit from glutamine supplementation [30,31]. In a previous ex vivo study, we have shown that glutamine did not induce mRNA HSP72 in critically ill patients but modified intracellular HSP72 after lipopolysaccharide (LPS) or HS induction unpredictably [32]. In an animal pancreatitis model, pre-treatment with parenteral glutamine increased lung and liver HSP90 expression and improved various inflammatory responses [33]. Another study in burned rats showed that parenteral glutamine supplementation combined with enteral nutrition upregulated the HSP90 expression, attenuated Peyer’s patch apoptosis, and improved intestinal IgA response [34]. 

Human peripheral blood mononuclear cells (PBMC) are the mainstay of immunology owing to stability, flexibility, isolation, and handling for numerous blood assays [5]. An ex vivo model with human PBMCs has been previously implemented to study the host–stimulant interaction in specific infections [4]. In a recent study, monocyte-accentuated innate immune responses to infection of human PBMCs exposed to SARS-CoV-2 in ex vivo conditions resembled those detected in vivo in patients with mild COVID-19 [6]. Likewise, glutamine’s protective effects were studied in a rat clonal b-cell line via specific pathways following ex vivo stimulation with cytokines derived from human PBMCs exposed to LPS [35]. Examining the time–dose effects of 5 or 10 mM L-alanine-glutamine (AL-GLn) and compared with those of 5 or 10 mM L-glutamine in PBMCs of septic patients incubated with 1 or 10 μg/mL LPS for 0, 4, or 24 h, we have previously shown that 10 mM L-Ala-Gln suppresses HSP72 and does not induce any of the T helper cells type 1 (Th1), Th2, and Th17 [36]. The present study aimed to investigate the effect of glutamine added to incubation media on HSP90α and cytokine release after LPS or HS induction in an ex vivo model using PBMCs from ICU patients with septic shock compared to severe trauma patients with SIRS and healthy volunteers.

## 2. Materials and Methods

### 2.1. Subjects

Critically ill patients diagnosed with septic shock (*n* = 11) or trauma-related SIRS (*n* = 10) and healthy subjects (*n* = 19) participated in this study. The study was approved by the Hospital Ethics Committee of Evangelismos Hospital (reference ID 41-09/02/2012), and written informed consent was obtained from patients’ relatives and healthy volunteers. Lactate levels > 2 mmol/L and vasopressor support to maintain mean arterial pressure > 65 mmHg were the criteria used to identify patients with septic shock [37]. Patients with severe trauma who met at least two of the four conventional SIRS criteria comprised the non-infectious ICU group [38].

### 2.2. Sample Collection

Twenty mL blood was drawn between 8:00 and 9:00 a.m. within 48 h of diagnosis in a sterile heparinized tube. If available, one or two control samples were drawn and processed simultaneously with every patient sample. We processed the blood within 1 h after collection.

### 2.3. Cell Culture Protocol

We aimed to estimate the pre-treatment and the post-treatment effects of glutamine on the induction or repression effects of LPS or HS on HSP90α mRNA, HSP90α intracellular proteins, and supernatant cytokine expression of PBMCs’ in sepsis compared to trauma-related SIRS and healthy subjects. First, we isolated fresh PBMCs from intensive care unit (ICU) patients with septic shock or trauma with SIRS and healthy controls by gradient centrifugation as previously described [36]. Twenty mL of RPMI 1640 (Gibco™, Invitrogen, Paisley, UK) added to the blood sample and the mixture overlaid to 10 mL Biocoll (Biochrom, Berlin, Germany). Then, the mixture was centrifuged at 300× *g* for 30 min at room temperature and the PBMC layer was transferred to a new tube (50 mL) and washed twice with RPMI 1640 at 300× *g* for 10 min. We counted cell concentration and viability by single platform flow cytometry analysis using the following combination: CD45 FITC/CD3 PE/7-Aminoactinomycin (Beckman Coulter, Immunotech, Marseille, France), along with absolute count fluorospheres (fluorescent microbeads, Beckman Coulter, Immunotech, Marseille, France). Viability was tested in each intervention before the cell culture and afterwards to verify that the cells survived. We incubated the cells at a concentration of 10^6^ cells/mL in Roswell Park Memorial Institute glutamine-free medium containing 5% fetal bovine serum and 1% penicillin/streptomycin (Gibco, Life Technologies, Carlsbad, CA, USA) in a humidified incubator containing 5% carbon dioxide (CO_2_) at 37 °C using 96-well culture plates. Following that, cells were either (a) stimulated with LPS Solution (final concentration 1 μg/mL) (O111:B4, Sigma™, St. Louis, MO, USA), or (b) submitted to/underwent heat shock (30 min in 43° water bath), or (c) left untreated. We selected 43 °C (HS), which has been shown to reduce the expression of human tissue factor-specific mRNA surface protein and activity induced by LPS stimulation [39].

We added 10 mM L-Ala-Gln (Dipeptiven™, Fresenius-Kabi, Bad Homburg, Germany) (a) 1 h before stimulation; (b) 1 h after stimulation; and (c) never. We chose the 10 mM L-Ala-Gln dose because (a) it corresponds to 10 mM plasma concentration at the recommended infusion rate of 0.75 g/kg L-Ala-Gln [40], and (b) at this dose, it induces maximal HSP expression, attenuating cytokine release [41]. In experimental studies, increasing glutamine dose from 2 to 10 mM decreased chemokines in human epithelial cell lines [3] and reduced the intestinal inflammatory response during cytokine stimulation [2]. Having accomplished the described HS, LPS, and glutamine interventions, cells were incubated for 4 h at 37 °C and harvested for intracellular staining and flow cytometric HSP90α analysis. The incubation period was set at 4 h because, in our pilot studies, 10 mM L-Ala-Gln was shown to repressed HSP72 by 4 h [36]. We selected an LPS dose of 1 μg/mL because, in the same timing-escalating dose–response trial, a 1 μg/mL LPS dose was shown to induce different interleukin responses at 24 h in PBMCs of patients and healthy controls [36].

### 2.4. HSP90α Flow Cytometry

Cells were stained for surface antigens CD33 PE/Cy5 and CD45 PE/Cy7 (clones WM33 and HI30, respectively, both from BioLegend, San Diego, CA, USA), fixed with paraformaldehyde, permeabilized with saponin (Beckman Coulter, Immunotech, Marseille, France), and incubated with phycoerythrin (PE)—conjugated anti-HSP90a monoclonal antibodies (Enzo Life Sciences, Ann Arbor, MI, USA) at a 1:10 (*v/v*) dilution. Flow cytometric analysis was performed (a) within 1 h of blood sampling (subjunctive’s baseline) and (b) 4 h after induction on an FC-500 instrument (Beckman Coulter, Miami, FL, USA) using CXP software version 2.2 (Beckman Coulter, Miami, FL, USA). Identification of PBMCs was achieved by the expression of CD45 compared to sideward scattering. Monocyte (CD33 ^++^ CD45 ^+^ SS ^med^) and lymphocyte (CD33 ^−^ CD45 ^++^ SS ^low^) populations were further identified based on CD33 and CD45 expression intensity. HSP90 intracellular expression was assessed in each cell type using mean fluorescence intensity (MFI). We performed instrumental quality control routinely to adjust acquisition settings to any photomultiplier (PMT) voltage alterations.

### 2.5. Gene Expression Assays

#### 2.5.1. The Gene Expression Assays Have Been Previously Described in Detail [32]

**Figure 1:**
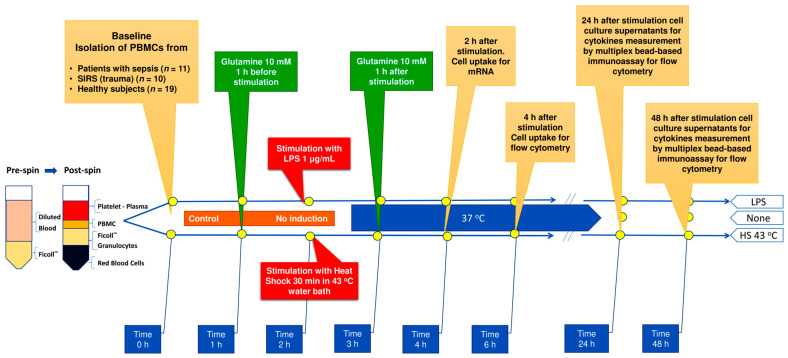
Experimental design. Abbreviations: PBMCs = peripheral blood mononuclear cells; SIRS = systemic inflammatory response syndrome; mRNA = messenger ribonucleic acid; LPS = lipopolysaccharide; HS = heat shock; Pre-spin = before centrifugation; Post-spin = after centrifugation at 300× *g* for 30 min.

Specifically, we seeded PBMCs (1 × 10^6^) in RPMI-1640 glutamine-free medium (1 mL, with fetal bovine serum (FBS) 10% and penicillin/streptomycin 1%) in cell culture 24-well plates. The experimental workflow design is depicted in Figure 1. (A) LSP assay: (A1) no LPS; (B2) LPS (*Escherichia coli* O111:B4, Sigma, St. Louis, MO, USA) 1 μg/mL; (A3) glutamine (L-Ala-Gln dipeptide (Dipeptiven; Fresenius-Kabi, Bad Homburg, Germany) 10 mM pre-treatment followed by LPS 1 h later; (A4) glutamine post-treatment, given 1 h after LPS. (B) Heat shock assay: B1) no stimulation, (B2) 43 °C heat shock 30 min duration; (B3) glutamine pre-treatment followed by HS 1 h later; (B4) glutamine post-treatment, given 1 h after HS. We ran all stimulations in a humidified incubator containing 5% CO_2_ at 37 °C and harvested cells 2 h after the final stimulation.

#### 2.5.2. RNA Isolation from PBMC

We harvested PBMCs from the wells to 1.5 mL microcentrifuge tubes and centrifuged at 360× *g* for 5 min. Then, we discarded supernatant and homogenized cells with 750 μL TRIzol reagent (Ambion, Carlsbad, CA, USA), separated RNA with chloroform, and precipitated with isopropanol. Following that, we washed the RNA with 75% alcohol and resuspended it in prewarmed, nuclease-free water (H_2_O). We measured the RNA concentration with Qubit RNA Assay Kit (Invitrogen, Eugene, OR, USA) on the Qubit 2.0 Fluorometer (Invitrogen, Eugene, OR, USA). Finally, we confirmed RNA integrity by agarose gel electrophoresis. To diminish possible genomic DNA contamination, we treated all total RNA samples with DNase I, Amplification Grade (Invitrogen, Eugene, OR, USA).

#### 2.5.3. Reverse Transcription and Quantitative PCR

Following the manufacturer’s instructions, 100 ng of total RNA was reverse transcribed with Transcriptor High Fidelity cDNA Synthesis Kit (Roche Diagnostics GmbH, Mannheim, Germany). With total RNA, we used 60 μM random hexamer primers, 1 mM each of dNTPs, 1× reaction buffer, 20 U RNase inhibitor, and 10 U of Transcriptor High Fidelity Reverse Transcriptase in a total volume of 20 μL. The thermal cycling conditions were 25 °C for 10 min, 60 °C for 60 min, and 85 °C for 5 min.

#### 2.5.4. Complementary DNA (cDNA)

In a total volume of 20 μL, cDNA was amplified by polymerase chain reaction (PCR) using 1× Maxima SYBR Green qPCR Master Mix (Thermo Scientific, Vilnius, Lithuania) and 0.2 μM of each primer. For the HS 90 kDa protein α (gene symbol HSP90α, amplicon size (bp) 234) the following sequences of the primers were used: 5′-3′F:GGCAGAGGCTGATAAGAACG; R:CGTGATGTGTCGTCATCTCC. Reaction mixtures were incubated in a Biorad CFX96, C1000 thermal cycler (ΒΙO-RAD, Hercules, CA, USA) at specific thermal conditions: initial denaturation at 95 °C for 5 min followed by 40 cycles of 95 °C for 15 s and 63 °C for 60 s. We measured fluorescence emitted by SYBR Green at the end of each cycle and determined nonspecific amplification by a melting curve analysis following cDNA amplification. Then we calculated the relative quantification of the target genes by the delta-delta CT (ΔΔCT) method using β-2 microglobulin (B2M) as a reference gene.

### 2.6. Supernatant Cytokines

We collected supernatants of cell cultures 24 and 48 h after stimulation and kept them at −80 °C. We measured supernatant IL-1β, IL-6, IL-8, IL-10, TNF-α, and MCP-1 levels using a multiplex bead-based immunoassay for flow cytometry (Cytometric Bead Array BD Biosciences, San Jose, CA, USA). Namely, we incubated samples with a mix of antibody-coated fluorescent beads and measured proteins using fluorochrome-conjugated monoclonal antibodies. We analyzed samples using the FCAP Array v3.0 Software (FACS Array, Becton Dickinson, BD Biosciences, San Jose, CA, USA) in a dual-LASER flow cytometer (FACS Array, Becton Dickinson, BD Biosciences, San Jose, CA, USA).

### 2.7. Serum Cytokines

We measured serum IL-6, IL-10, IL-17, and interferon-γ (IFN-γ) concentrations by ELISA assay according to the manufacturer’s instructions (Invitrogen, Carlsbad, CA, USA). The inter-assay and intra-assay coefficient of variation (CV) for each analyte were as follows: 6.2 and 7.8 pg/mL for IL-6, 3.25 and 2.75 pg/mL for IL-10, 3.7 and 3.5 for IL-17 pg/mL, and 7.3 and 7.1 pg/mL for IFN-γ. The sensitivities of the assays were <2 pg/mL for IL-6, <1 pg/mL for IL-10, 2 pg/mL for IL-17, and 0.03 IU/mL for IFN-γ.

### 2.8. Statistical Analysis

The Shapiro–Wilk test was used to assess the normality of the distribution. Results are presented as mean ± standard error (SE) of mean and median (interquartile range) as appropriate. Categorical variables were analyzed using the *x^2^* test. Comparisons among groups for continuous variables were carried out by one-way ANOVA with Tukey’s honestly significant differences (HSD) as a post hoc test for parametric data. The independent-samples Kruskal–Wallis one-way analysis of variance by ranks across groups was used for nonparametric data, adjusted by the Bonferroni correction for multiple tests. Paired differences for continuous variables in the same subjects were analyzed using the related samples of Friedman’s two-way analysis of variance by ranks. Pairwise comparisons between pairs of no stimulation and stimulation with or without glutamine before or after LPS or HS were analyzed using the Wilcoxon signed-ranks test, adjusted by the Bonferroni correction for multiple tests. A two-sided significance level of 0.05 was used for statistical inference. We used the IBM SPSS software platform (v.28, IBM SPSS Statistics, Chicago, IL, USA) for statistical analyses and the GraphPad Prism 9.0 (GraphPad Software, Inc., San Diego, CA, USA) for additional statistics and illustrations.

## 3. Results 

### 3.1. Study Population 

Demographic and clinical characteristics are shown in Table 1. Groups did not differ regarding age or sex and the Sequential Organ Failure Assessment (SOFA). Sepsis (all with septic shock) and SIRS patients differed significantly regarding the outcome, the Acute Physiology and Chronic Health Evaluation II (APACHE II) score, and temperature. Patients with sepsis had increased serum levels of IL-6, IL-10, IFN-γ, and neutrophil HSP90α expression compared to controls but repressed monocyte HSP90α. Despite the increased serum levels of cytokines in sepsis compared to SIRS, only serum IL-6 reached statistical significance (*p* < 0.001).

### 3.2. Differences and Glutamine Effect in Relative HSP90α Gene Expression 

Relative HSP90α gene expression did not differ among groups at baseline or after LPS stimulation but increased significantly after HS induction, especially in sepsis (*p* < 0.001) (Table 2). 

**Figure 2:**
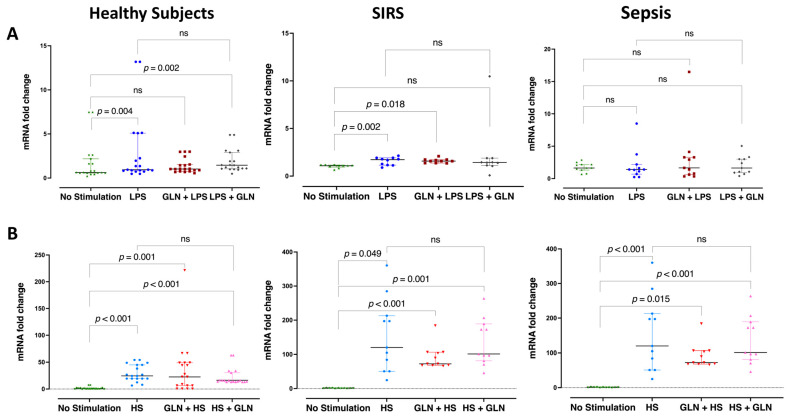
Glutamine effect on heat shock protein 90α (HSP90α) relative mRNA expression in peripheral blood mononuclear cells (PBMCs) within 2 h of LPS or HS stimulation. (**A**) LPS increased the HSP90α mRNA expression in the PBMCs of healthy subjects and patients with SIRS (trauma) but not in patients with sepsis. Glutamine 1 h before (GLN + LPS) or after (LPS + GLN) stimulation did not alter any induction effect of LPS on the mRNA gene expression of PBMCs in all groups. (**B**) HS increased the HSP90α mRNA expression in the PBMCs in all groups, almost 10-fold in sepsis compared to the other groups. Glutamine 1 h before (GLN + HS) or after (HS + GLN) stimulation did not alter any induction effect of HS on the mRNA gene expression of PBMCs in all groups Abbreviations: SIRS = systemic inflammatory response syndrome; mRNA = messenger ribonucleic acid; LPS = lipopolysaccharide; GLN = glutamine; HS = heat shock; ns = not significant. Each dot represents an individual patient; dots of different colors represent different stimulant situations as indicated in axis labels; lines represent medians and bars interquartile ranges. There were no missing data; pairwise comparisons were tested using the related samples Friedman’s two-way analysis of variance by ranks; *p*-values were determined using the Wilcoxon signed-ranks test between pairs of no stimulation and stimulation with or without GLN before or after LPS or HS, adjusted by the Bonferroni correction for multiple tests.

LPS increased the HSP90α mRNA expression in healthy subjects (*p* < 0.001) and SIRS (*p* = 0.002) but not in sepsis (Figure 2A). HS increased the HSP90α mRNA expression in the PBMCs in all groups (*p* < 0.001) and almost 10-fold in sepsis compared to the other groups (Figure 2B). Glutamine before or after stimulation did not alter any induction effect of either LPS or HS on the mRNA gene expression of PBMCs in all groups (Figure 2A,B).

### 3.3. Differences and Glutamine Effect in Intracellular Monocyte HSP90α Protein Expression

Monocyte HSP90α protein expression was increased at baseline (*p* = 0.008) or after LPS stimulation (*p* = 0.021) in septic and trauma patients compared to healthy subjects and after HS induction in septic patients compared to healthy patients (*p* = 0.005) (Table 3). 

**Figure 3:**
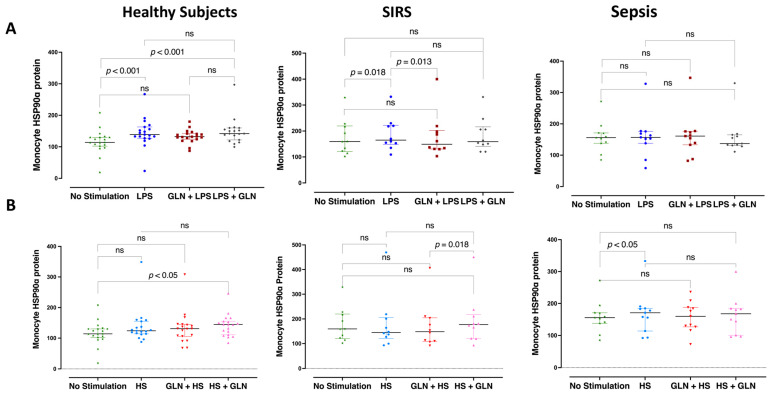
Glutamine effect on heat shock protein 90α expression in monocytes (mHSP90α) within 4 h of LPS or HS stimulation. (**A**) LPS increased the mHSP90α expression in the PBMCs of healthy subjects and patients with SIRS (trauma) but not in patients with sepsis. Glutamine before LPS decreased the induced mHSP90α protein expression in SIRS but increased it in healthy subjects when given after LPS. Either way, glutamine did not affect the monocyte HSP90α protein expression in sepsis. (**B**) HS increased the mHSP90α expression in the PBMCs in all groups, almost 10-fold in sepsis compared to the other groups. Glutamine before or after HS stimulation showed a non-significant decreasing trend in sepsis but a significant increase in SIRS and healthy subjects when given after HS induction. Abbreviations: SIRS = systemic inflammatory response syndrome; HSP = heat shock protein; mHSP90α = monocyte HSP90α protein; LPS = lipopolysaccharide; HS = heat shock; ns = not significant. Each dot represents an individual patient; dots of different colors represent different stimulant situations as indicated in axis labels; lines represent medians and bars interquartile ranges. There were no missing data; pairwise comparisons were tested using the related samples Friedman’s two-way analysis of variance by ranks; *p* values were determined using the Wilcoxon signed-ranks test between pairs of no stimulation and stimulation with or without GLN before or after LPS or HS, adjusted by the Bonferroni correction for multiple tests.

LPS increased the monocyte HSP90α protein expression in healthy subjects (*p* < 0.001) and SIRS (*p* = 0.018) but not in sepsis (Figure 3A). HS increased the monocyte HSP90α protein expression only in sepsis (*p* = 0.047) (Figure 3B). Glutamine before LPS decreased the induced monocyte HSP90α protein expression in SIRS (*p* = 0.013) but retained it in healthy subjects when given after LPS *(p* < 0.001). Either way, glutamine did not affect the monocyte HSP90α protein expression in sepsis (Figure 3A). Glutamine before or after HS stimulation resulted in a non-significant decreasing HSP90α trend in sepsis but a significant increase in SIRS compared to pre-treatment (*p* = 0.018) and healthy subjects compared to baseline (*p* < 0.05) when given after HS induction (Figure 3B).

### 3.4. Differences and Glutamine Effect in Intracellular Lymphocyte HSP90α Protein Expression

Lymphocyte HSP90α protein expression was increased at baseline in septic and trauma patients compared to healthy subjects (*p* < 0.001). It was also increased after LPS (*p* < 0.001) or HS induction (*p* = 0.002) in septic patients compared to healthy and/or to SIRS (*p* = 0.002) (Table 4). 

**Figure 4:**
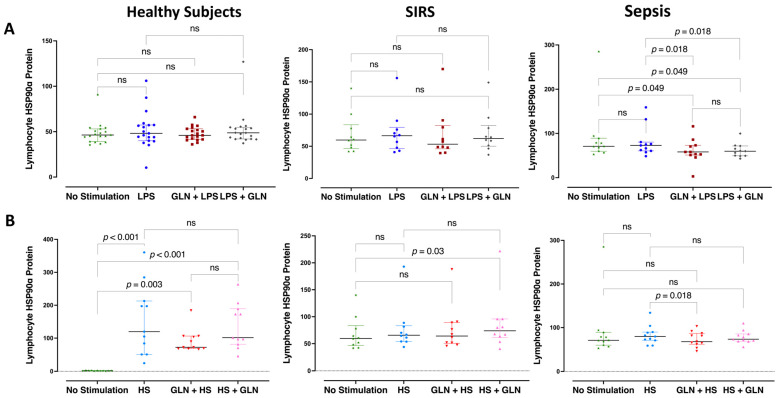
Heat shock protein 90α expression in lymphocytes (lHSP90α) within 4 h of LPS or HS stimulation. Lymphocyte HSP90α protein expression was increased at baseline in septic and trauma patients compared to healthy subjects (*p* < 0.001). (**A**) LPS stimulation did not increase the lymphocyte HSP90α protein expression in any group. Glutamine before or after LPS decreased the LPS-induced lHSP90α expression in sepsis. (**B**) HS increased the lHSP90α expression in the PBMCs in the control group only (*p* < 0.001). Glutamine before HS stimulation decreased the HS-induced lHSP90α expression in sepsis but retained it in healthy subjects and increased it in SIRS when given after HS stimulation. Abbreviations: SIRS = systemic inflammatory response syndrome; HSP = heat shock protein; lHSP90α = lymphocyte HSP90α protein; LPS = lipopolysaccharide; HS = heat shock; ns = not significant. Each dot represents an individual patient; dots of different colors represent different stimulant situations as indicated in axis labels; lines represent medians and bars interquartile ranges. There were no missing data; pairwise comparisons were tested using the related samples Friedman’s two-way analysis of variance by ranks; *p* -values were determined using the Wilcoxon signed-ranks test between pairs of no stimulation and stimulation with or without GLN before or after LPS or HS, adjusted by the Bonferroni correction for multiple tests.

LPS stimulation did not increase the lymphocyte HSP90α protein expression in any group, whereas HS increased it in the control group only (*p* < 0.001) (Figure 4A,B). Glutamine before or after LPS (*p* = 0.049) (Figure 4A) and before HS stimulation (*p* = 0.018) decreased the lymphocyte HSP90α expression in sepsis. In contrast, glutamine retained the HS-induced increased lHSP90α expression in healthy subjects (*p* < 0.001) and increased it in SIRS when given after HS stimulation (*p* = 0.03) (Figure 4B).

### 3.5. Group Differences in Supernatant Cytokine Level

Before stimulation, supernatant cytokine levels did not differ among groups, except MCP-1, which was significantly repressed at 48 h in sepsis and SIRS compared to healthy subjects (*p* = 0.002). After LPS induction, supernatant IL-6 (*p* = 0.005), IL-8 (*p* = 0.01), IL-10 (*p* = 0.001), and MCP-1 (*p* = 0.029) at 24 h and IL-8 (*p* = 0.015), IL-10 (*p* < 0.001), and MCP-1 (*p* < 0.001) at 48 h were higher in SIRS compared to healthy control and/or sepsis (Table 5). Compared to healthy subjects, LPS induced MCP-1 in PBMCs of patients with sepsis at 48 h (*p* < 0.001) but repressed IL-10 at 24 (*p* = 0.001) and 48 h (*p* < 0.001). HS stimulation only induced supernatant cytokine IL-8 in SIRS compared to healthy and sepsis at 48 h (*p* = 0.068), but this difference did not reach statistical significance (Table 5).

### 3.6. Induction Differences and Glutamine Effect in Supernatant Cytokine Levels

**Figure 5:**
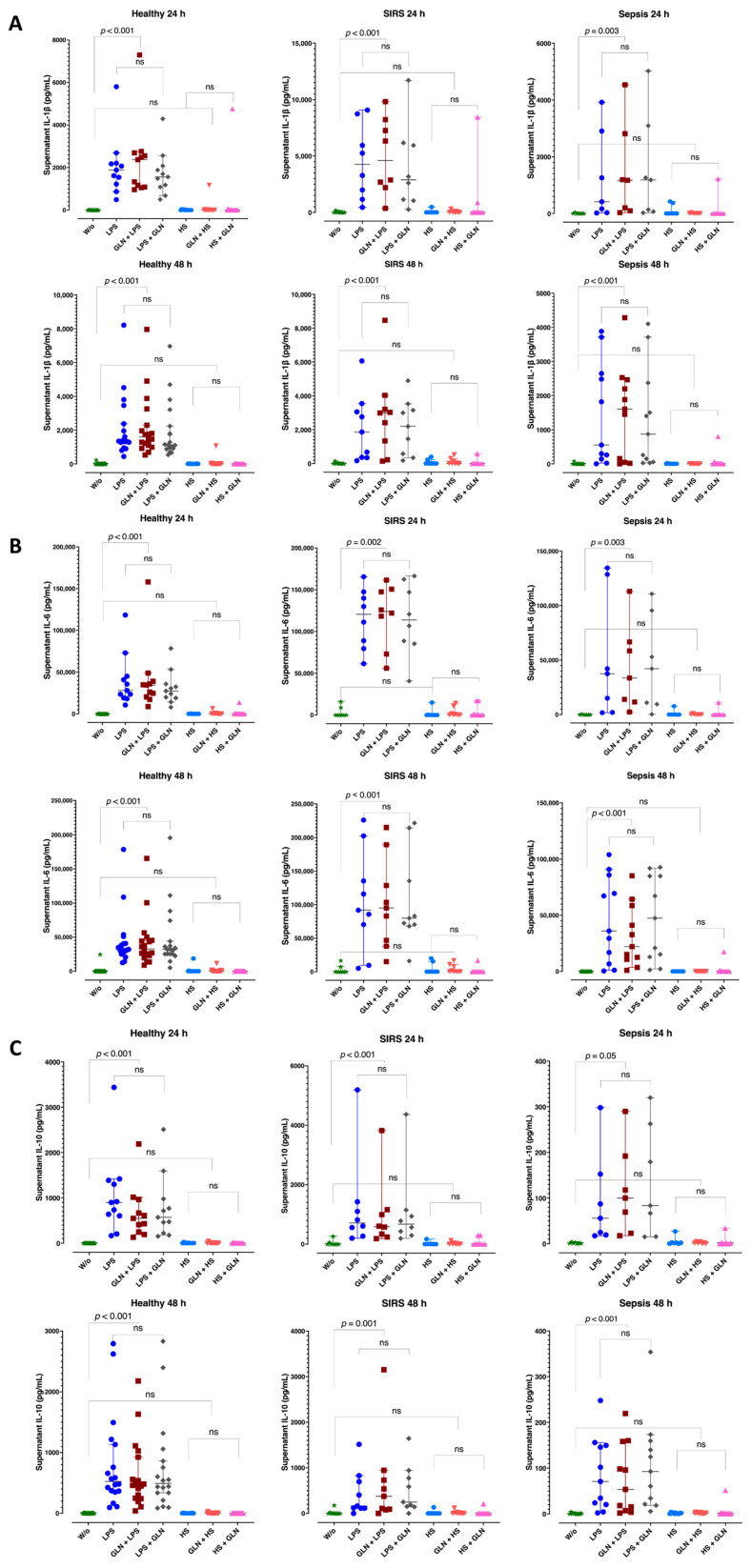
Supernatant IL-1β, IL-6, and IL-10 at 24 and 48 h of LPS or HS stimulation. Before stimulation, supernatant interleukins did not differ among groups. LPS induction increased the supernatant IL-1β (**A**), IL-6 (**B**), and IL-10 (**C**) concentrations at 24 and 48 h in all groups. LPS-induced interleukins did not differ between healthy and sepsis but were higher in SIRS. HS did not induce IL-1β, IL-6, and IL-10 in any group. Glutamine pre- or post-treatment neither repressed the LPS induction nor enhanced the insignificant HS effect in IL-1β, IL-6, and IL-10 supernatant concentrations (**A–C**). Abbreviations: SIRS = systemic inflammatory response syndrome; IL = interleukin; LPS = lipopolysaccharide; HS = heat shock; ns = not significant; W/o = without stimulation. Each dot represents an individual patient; dots of different colors represent different stimulant situations as indicated in axis labels; lines represent medians and bars interquartile ranges. There were missing data at 24 h; pairwise comparisons were tested using the related samples Friedman’s two-way analysis of variance by ranks; *p* values were determined using the Wilcoxon signed-ranks test between pairs of no stimulation and stimulation with or without GLN before or after LPS or HS, adjusted by the Bonferroni correction for multiple tests.

LPS induction increased the supernatant IL-1β (Figure 5A), IL-6 (Figure 5B), and IL-10 (Figure 5C) concentrations at 24 and 48 h in healthy subjects, trauma, and septic patients (*p* < 0.001). HS did not induce the interleukins in any group. Glutamine pre- or post-treatment neither repressed the LPS induction nor enhanced the insignificant HS effect in IL-1β, IL-6, and IL-10 supernatant concentrations (Figure 5A–C). 

**Figure 6:**
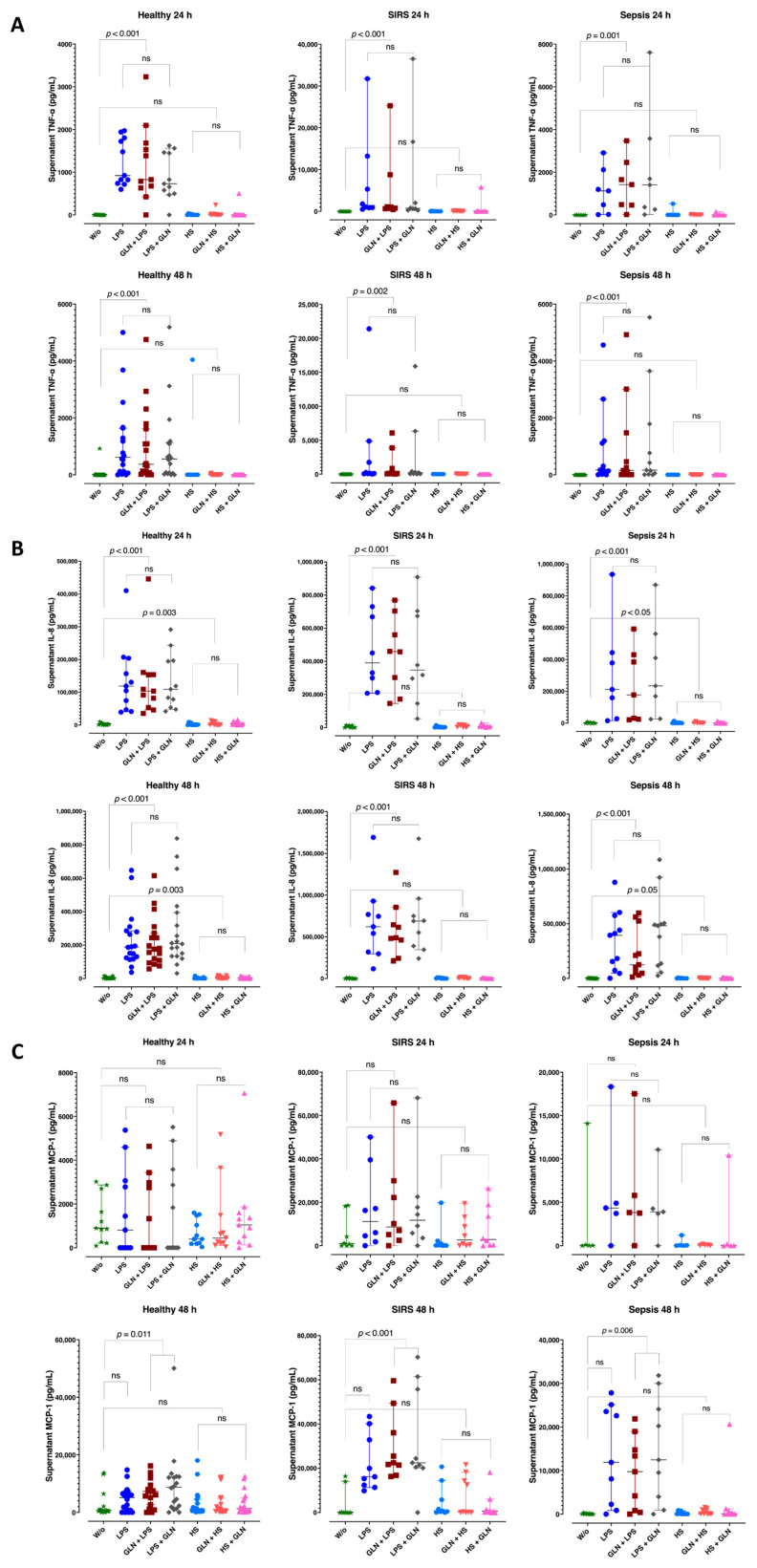
Supernatant TNF-α, IL-8, and MCP-1 at 24 and 48 h of LPS or HS stimulation. Before stimulation, supernatant cytokines did not differ among groups. (**A**) LPS induction increased the supernatant TNF-α concentrations at 24 and 48 h in all groups. HS did not induce TNF-α in any group. Glutamine pre- or post-treatment neither repressed the LPS induction nor enhanced the insignificant HS effect in TNF-α supernatant concentrations. (**B**) LPS increased IL-8 concentrations at 24 and 48 h in all groups. HS induced the supernatant IL-8 in healthy and septic groups at 24 and 48 h. Glutamine pre- or post-treatment did not repress the LPS or HS induction effect in IL-8 supernatant concentrations. (**C**) Neither LPS nor HS induced the supernatant MCP-1 at 24 or 48 h in any group. Glutamine pre- or post-treatment increased the LPS induction effect in MCP-1 supernatant concentrations in healthy and SIRS and post-treatment in sepsis at 48 h. Glutamine did not alter the insignificant HS effect. Abbreviations: SIRS = systemic inflammatory response syndrome; IL = interleukin; TNF-α = tumor necrosis factor-α; MCP-1 = monocyte chemoattractant protein-1; LPS = lipopolysaccharide; HS = heat shock; ns = not significant; W/o = without stimulation. Each dot represents an individual patient; dots of different colors represent different stimulant situations as indicated in axis labels; lines represent medians and bars interquartile ranges. There were missing data at 24 h; pairwise comparisons were tested using the related samples Friedman’s Two-way analysis of variance by ranks; *p* -values were determined using the Wilcoxon signed-ranks test between pairs of no stimulation and stimulation with or without GLN before or after LPS or HS, adjusted by the Bonferroni correction for multiple tests.

LPS induction increased the supernatant TNF-α (Figure 6A) at 24 and 48 h in healthy subjects, trauma, and septic patients. HS did not induce TNF-α in any group. Glutamine pre- or post-treatment neither repressed the LPS induction nor enhanced the insignificant HS effect in TNF-α supernatant concentrations (Figure 6A). 

LPS increased IL-8 concentrations at 24 and 48 h in all groups. HS induced the supernatant IL-8 concentrations in the healthy and septic groups at 24 and 48 h (Figure 6B). Glutamine pre- or post-treatment did not repress the LPS or HS induction effect in IL-8 supernatant concentrations.

Neither LPS nor HS induced the supernatant MCP-1 at 24 or 48 h in any group (Figure 6C). Glutamine pre- or post-treatment increased the LPS induction effect in MCP-1 supernatant concentrations in healthy (*p* = 0.011), SIRS (*p* < 0.001) and sepsis (*p* = 0.006) at 48 h (Figure 6C). Glutamine did not alter the insignificant HS effect. 

## 4. Discussion 

Our results show that heat shock increases the HSP90α mRNA and the monocyte HSP90α protein expression in the PBMCs in all groups (10-fold in patients with septic shock). Regarding cytokines, HS induces only the supernatant IL-8 in healthy and septic subjects. We showed that LPS does not increase the expression of HSP90α mRNA and mHSP90α or lHSP90α protein in sepsis but induces them in healthy subjects and patients with SIRS (trauma). In contrast, LPS increases the supernatant IL-1β, IL-6, IL-8, IL-10, and TNF-α concentrations at 24 and 48 h in the healthy and critically ill, especially in trauma patients. Our data confirm that the glutamine at 10 mM before or after stimulation does not alter any induction effect of either LPS or HS on relative HSP90α gene expression and the monocyte HSP90α protein of PBMCs in sepsis. In trauma-related SIRS, however, glutamine before LPS decreases the mHSP90α and increases it when given after HS. We also showed that before or after LPS and before HS, glutamine decreases the lymphocyte HSP90α expression in sepsis but increases it when given after HS in SIRS. Finally, we showed that glutamine does not alter the LPS or HS effect on IL-1β, IL-6, IL-8, IL-10, and TNF-α supernatant concentrations but enhances the LPS-induced MCP-1 in all groups.

A novel finding of our study is that the glutamine effect is complex depending on timing (pre-treatment or post-treatment), biomolecule target (HSP90α mRNA, intracellular proteins, interleukins, MCP-1), cell type (monocytes or lymphocytes), and patients’ sub cohorts (sepsis or trauma), being different in patients with septic shock or SIRS and healthy subjects. In PBMCs of septic patients, HSP90α is promptly induced by HS but not by LPS, whereas cytokines are poorly induced compared to trauma patients. Except for MCP-1, which is enhanced, glutamine does not modulate any other cytokine. The glutamine pre-treatment repressing effect on lHSP90α in sepsis might be related to a high glutamine consumption of the rapidly dividing lymphocytes, expressing an increased need for nucleotide biosynthesis [42]. Similarly, monocytes express high glutamine consumption rates to meet critical functions in an emergency [42]. In trauma, glutamine decreases the induced mHSP90α before LPS but increases mHSP90α and lHSP90α when given after HS. These findings might explain the disappointing results of prospective randomized studies challenging previous guidelines for enteral and parenteral glutamine supplementation in critically ill patients, as safety concerns have been raised [43]. In agreement with our results, current European Society for Clinical Nutrition and Metabolism (ESPEN) [44], European Society of Paediatric and Neonatal Intensive Care (ESPNIC) [45], and American Society for Parenteral and Enteral Nutrition (ASPEN) [46] guidelines do not recommend glutamine supplementation in critically ill patients. Future research should examine the impact of glutamine supplementation and temperature modulation on HSP90α and MCP-1 pathways in sepsis and trauma.

What is characteristic in our series is that patients with septic shock were in a hyperinflammatory state since they had increased serum levels of IFN-γ, IL-6, and IL-10 compared to controls and IL-6 compared to SIRS. The increased neutrophil HSP90α expression in our septic patients might be related to the inhibition of the catalytic activity of caspases-8 and -3 [47] by stabilizing a molecular complex of c-Src kinase and caspase-8, prolonging the survival of activated neutrophils [48]. Additionally, since heat shock causes rapid translocation of IkappaB kinase (IKK) and nuclear factor kappa B (NF-κB) complexes into the plasma-membrane-associated lipid rafts and segregates the HSP90α cofactor from the IKK complex, it might contribute to the apoptotic loss of lymphocytes in sepsis-associated hyperthermia [49]. This mechanism might explain our finding that heat shock increases the HSP90α mRNA and the monocyte HSP90α protein expression in the PBMCs, especially in sepsis. Regarding cytokines, however, we found that HS does not induce any other cytokine except IL-8 in healthy and septic subjects. In cells stimulated with cytokine, 193 genes change expression, 86 genes are modulated similarly by cytokine or heat shock, while HS pre-treatment affects the expression of 2/3 of cytokine-modulated genes [50]. Thus cell exposure to HS (43 °C 1 h) inhibited the NF-κB signaling response to TNF-α and IL-1β stimulation, although uptake of cytokines was not impaired [51]. 

In our study, septic patients had repressed monocyte HSP90α, whereas LPS did not increase the HSP90α mRNA expression. These results expand on findings of a previous study, showing a repressed mean fluorescent intensity of monocyte and neutrophil HSP90α in septic shock associated with a repressed CD14/HLA-DR expression [52]. In contrast, LPS increased the supernatant IL-1β, IL-6, IL-8, IL-10, and TNF-α concentrations at 24 and 48 h better in patients with trauma than sepsis. Decreased natural killer (NK)-cell activity and cytokine release from LPS-stimulated PBMCs indicate hypo-responsiveness of NK cells and impaired early inflammatory response in critical illness [53]. It has been previously shown that prior exposure of PBMCs to bacterial ligands alters macrophage cytokine production in response to subsequent LPS-stimulated activation since PBMCs develop a repressed state of LPS-responsiveness, the “LPS tolerance” [54]. LPS tolerance was associated with the inhibition of cytokines release and decreased extracellular signal-regulated kinase and p38 kinase activation when macrophages were restimulated with LPS [54].

Our data confirm that glutamine at 10 mM before or after stimulation cannot modulate any induction effect of either LPS or HS on relative HSP90α gene expression, monocyte HSP90α protein, and supernatant IL-1β, IL-6, IL-8, IL-10, and TNF-α concentrations of PBMCs in sepsis. In a randomized, double-blind, interventional clinical trial in critically ill children with sepsis or surgery, glutamine supplementation maintained HSP70 levels for longer but did not increase glutamine levels and did not influence IL-10 or IL-6 levels and outcome [55]. Addition of 2 mM glutamine to the incubation media enhanced extracellular HSP70, TNF-α, and IL-10 release at 4 h and IL-8 at 24 h [56,57]. Increased release of extracellular HSPs was associated with repressed intracellular expression of HSP70 and HSP90α in sepsis [58,59,60]. As plasma from whole blood has been used in previous studies, discrimination of HSP70 released from granulocytes, lymphocytes, or peripheral mononuclear cells could not be established [56,57]. 

In an animal model, 12 h after LPS administration, IL-6, IL-8, and IL-1β concentrations and IL-1β, IL-8, TNF-α, and IL-6 gene expression were increased and attenuated by glutamine pre-treatment [61]. In addition, in the treated mice, glutamine alleviated LPS-induced pathological changes in the epithelial surface area of the airway and lung tissue. In a randomized alanyl-glutamine against LPS-induced lung injury study in adult Wistar rats, pre-administration of glutamine just before LPS could effectively protect the lung by enhancing HSP70 expression and decreasing plasma TNF-α, IL-1β, and IL-8 but delayed administration did not protect against LPS-induced lung injury [62]. In agreement with our results, these studies verify that glutamine administered after sepsis cannot ameliorate inflammation. Additionally, inhibition of NF-κB at the onset of sepsis may result in a decreased inflammatory response, but suppression of NF-κB afterwards may protract the inflammatory reaction [63]. Because sepsis represses the intracellular HSP70 and HSP90α [52,59], the activity of NF-κB cannot be degraded. In piglets, glutamine pre-treatment decreased serum TNF-ɑ and IL-6 and suppressed intestinal inflammation by modulating the crosstalk between adenosine 5′-monophosphate-activated protein kinase (AMPK) activation and mitochondrial function [64]. However, in another animal model, glutamine-treated sheep had increased myocardial HSPs following but not before endotoxemia [65]. 

In endotoxin-stimulated blood, there were no significant differences in the release of IL-6, TNF-α, and IL-1β with glutamine supplementation at 4 and 24 h. We have previously shown that glutamine does not induce any interleukin or chemokine of the Th1, Th2, and Th17 cells and suppresses HSP70 in human PBMCs [36]. We have also shown that the HSP90α time-series expression in healthy controls declines, contrasting the increasing trend of HSP70 but is increased in septic supernatants [32]. Hereby, we demonstrate that glutamine, before or after LPS and before HS, decreases the HSP90α expression in lymphocytes, and it induces the supernatant MCP-1 expression. High MCP-1 levels are associated with organ failure and poor outcome, while blocking MCP-1 synthesis protects mice from sepsis [66]. In a murine sepsis model, the LPS-induced MCP-1 in the lungs was significantly downregulated with the HSP90α inhibitor AUY-922 by counteracting main pro-inflammatory pathways [67].

### Limitations

Our ex vivo study provides further evidence for the failure of immunomodulatory effects of glutamine on human PBMCs in the context of HS- and LPS-induced HSP90α and cytokine responses in sepsis and trauma. The conclusions from our work should be regarded with some caution owing to certain limitations of the study, which should be addressed in future research. We investigated only one dose of glutamine (10 mM) that significantly upregulated HSP90α release in our LPS-glutamine time-dose pilot stimulation models [36]. Additionally, we used specific timing of pre- and post-treatment experiments and sampling schedules. It would be valuable to extend the research with different experimental conditions. Further, LPS represents a stimulus from Gram-negative bacteria, but sepsis can also be caused by other pathogens, including Gram-positive bacteria, viruses, and fungi. All these aspects are worthy of further investigation.

## 5. Conclusions

In the PBMCs of septic patients, mRNA, and monocyte HSP90α are promptly induced by HS but not by LPS, whereas cytokines are poorly induced compared to trauma patients. Glutamine, before or after LPS and before HS stimulation, decreases the lymphocyte HSP90α protein in sepsis. Before LPS, glutamine decreases the monocyte HSP90α in trauma but increases it when given before or after HS and the lymphocyte HSP90α when given after HS. Additionally, our data demonstrate that glutamine does not alter the LPS or HS effect in IL-1β, IL-6, IL-8, IL-10, and TNF-α concentrations and that post-treatment glutamine enhances the LPS effect in supernatant MCP-1 in all groups. Future research should seek to elucidate better the impact of glutamine and temperature modulation on HSP90α and MCP-1 pathways in sepsis.

## Figures and Tables

**Table 1:** Demographic and clinical characteristics.

Variables	Healthy Individuals	Patients with Trauma (SIRS)	Patients with Sepsis	^#^*p* Value
*n* = 40	*n* = 19	*n* = 10	*n* = 11	
Age (years)	39.3 ± 13	39.5 ± 15	49.2 ± 16	0.214
Sex (male/female)	7/12 (36.8/63.2)	5/6 (45.5/54.5)	4/7 (36.7/63.6)	0.877
APACHE II	-	10.6 ± 4.8	15.9 ± 4.8	**0.040**
SOFA	-	7.75 ± 3.2	9.3± 2.6	0.274
Temperature (°C)	-	36.4 ± 1.2	37.8 ± 1.1	**0.021**
mHSP90α (MFI)	80.5 (8; 90.5)	67.7 (59.9; 80.9)	55.5 (40.6; 58.5)	0.975
nHSP90α (MFl)	24.3 (5.0; 30.0) *	33.7 (21.0; 35.7)	40.2 (31.4; 41.9) *	**0.009**
IL-6 (pg/mL)	1.4 (1.0; 2.1) *	67.0 (45.4; 130.5) **	499.5 (82.3; 645.0) ***	**<0.001**
IL-10 (pg/mL)	7.7 (3.4; 13.7) *	19.4 (10.6; 26.6)	32.9 (15.7; 61.9) *	**<0.001**
IL-17 (pg/mL)	0.01 (0.01; 0.01)	0.86 (0.01; 5.96)	0.01 (0.01; 0.01)	0.557
IFN-γ (pg/mL)	4.85 (2.88; 7.44)	5.0 (4.3; 6.7)	45.0 (10.5; 144.5)	**<0.001**
Mortality	-	0 (0)	2 (5.1)	**0.047**

Continuous variables are reported as mean ± standard deviation for parametric data or 50th (median) and 25th and 75th percentiles (interquartile range, within brackets) for non-parametric data, as appropriate. Discrete variables are reported as the number and proportion (within brackets) of subjects with the characteristic of interest. Abbreviations: HSP = heat shock protein; mHSP90α = monocyte HSP90α protein expression; nHSP90α = neutrophil HSP90α protein expression; APACHE II = Acute Physiology and Chronic Health Evaluation II; SOFA = Sequential Organ Failure Assessment; MFI = mean fluorescence intensity; IL = interleukin; IFN = interferon; ^#^ Comparisons between groups were carried out by one-way ANOVA with Tukey’s honestly significant differences (HSD) as a post hoc test for parametric data and by independent samples Kruskal–Wallis one-way analysis of variance by ranks across groups for non-parametric data (significance values were adjusted by the Bonferroni correction for multiple tests; bold indicates statistical significant difference (*p* < 0.05); * Sepsis vs. Healthy; ** Sepsis vs. systemic inflammatory response syndrome (SIRS); *** SIRS vs. Healthy).

**Table 2:** Comparison analysis of relative HSP90α gene expression among different stimulation conditions of peripheral blood mononuclear cells without or with glutamine pre- or post-treatment in patients with sepsis or trauma and healthy subjects.

PBMCs from						Healthy Individuals	Patients with Trauma (SIRS)	Patients with Sepsis	
*n* = 40						*n* = 19	*n* = 10	*n* = 11	
Compared Conditions	Time (h)					HSP90α mRNA Fold Change			
	0	1	2	3	4	Median (IQR)	Median (IQR)	Median (IQR)	^#^*p* Value
Stimulation	None				mRNA	0.63 (0.56; 1.90)	1.08 (0.90; 1.14)	1.84 (1.31; 2.15)	0.052
			LPS		mRNA	0.95 (0.86; 3.67) ^^^^	1.67 (1.21; 1.85) ^^^^	1.37 (0.70; 2.18)	0.795
		GLN	LPS		mRNA	1.03 (0.83; 1.50)	1.58 (1.47; 1.70)	2.29 (0.61; 3.28)	0.155
			LPS	GLN	mRNA	1.46 (1.08; 2.40)	1.42 (1.13; 1.67)	1.72 (0.91; 3.00)	0.956
						^^^*p* < 0.001	^^^*p* = 0.002	^^^*p =* 0.819	
Stimulation	None				mRNA	0.63 (0.56; 1.90)	1.08 (0.90; 1.14)	1.84 (1.31; 2.15)	0.052
			HS		mRNA	24.59 (19.56; 44.98) ^^^^ *	19.03 (16.05; 44.98) ^^^^ **	112.32 (50.68; 197.88) ^^^^ * **	**<0.001**
		GLN	HS		mRNA	22.63 (6.36; 48.84) *	34.54 (31.02; 44.94) **	72.17 (67.81; 106.40) * **	**<0.001**
			HS	GLN	mRNA	16.45 (13.84; 23.68) *	23.92 (16.58; 32.34) **	137.10 (97.23; 189.14) * **	**<0.001**
						^^^*p* < 0.001	^^^*p* < 0.001	^^^*p* < 0.001	

Continuous variables are reported as median (interquartile range). Abbreviations: IQR = interquartile range; PBMCs = peripheral blood mononuclear cells; SIRS = systemic inflammatory response syndrome; LPS = lipopolysaccharide; HS = heat shock; GLN = glutamine; mRNA = messenger ribonucleic acid; HSP = heat shock protein; ^#^ independent samples Kruskal–Wallis one-way analysis of variance by ranks across groups (significance values were adjusted by the Bonferroni correction for multiple tests; bold indicates statistical significant difference (*p* < 0.05); * Sepsis vs. Healthy controls; ** Sepsis vs. SIRS); ^^^ related samples Friedman’s two-way analysis of variance by ranks; pairwise comparisons: Wilcoxon signed-ranks test (^^^^ *p* < 0.05 between pairs of no stimulation and stimulation with or without GLN before or after LPS or HS).

**Table 3:** Comparison analysis of monocyte HSP90α protein expression among different stimulation conditions with or without glutamine pre- or post-treatment in patients with sepsis or trauma and healthy subjects.

PBMCs from						Healthy Individuals	Patients with Trauma (SIRS)	Patients with Sepsis	
*n* = 40						*n* = 19	*n* = 10	*n* = 11	
Compared Conditions	Time (h)					Monocyte HSP90α Protein Expression			
	0	1	2	3	6	Median (IQR)	Median (IQR)	Median (IQR)	^#^*p* Value
Stimulation	None				mHSP90α	114.0 (104.0; 130.0) * ***	159.0 (128.5; 204.5) ***	156.0 (140.0; 170.5) *	**0.008**
			LPS		mHSP90α	139.0 (130.0; 159.5) ^^^^ * ***	170.0 (154.0; 219.0) ^^^^ ***	163.0 (155.0; 176.5) *	**0.021**
		GLN	LPS		mHSP90α	132.0 (126.0; 141.5)	161.0 (132.5; 192.0) ^^^^^	161.0 (136.0; 174.5)	0.066
			LPS	GLN	mHSP90α	142.0 (129.0; 157.5) ^^^^	164.0 (149.5; 206.0)	137.0 (132.0; 162.5)	0.177
						^^^*p* < 0.001	^^^*p* = 0.004	^^^*p* = 0.383	
Stimulation	None				mHSP90α	114.0 (104.0; 130.0) * ***	159.0 (128.5; 204.5) ***	156.0 (140.0; 170.5) *	**0.008**
			HS		mHSP90α	124.0 (115.0; 146.0) *	148.0 (132.0; 182.5)	183.0 (164.5; 191.5) ^^^^ *	**0.005**
		GLN	HS		mHSP90α	131.0 (107.0; 144.0)	153.0 (111.5; 189.0)	160.0 (128.5; 186.0)	0.161
			HS	GLN	mHSP90α	145.0 (116.5; 154.0) ^^^^	174.0 (143.5; 196.0) ^^^^^^	168.0 (123.0; 184.0)	0.123
						^^^*p* = 0.031	^^^*p* = 0.031	^^^*p* = 0.047	

Continuous variables are reported as median (interquartile range). Abbreviations: IQR = interquartile range; PBMCs = peripheral blood mononuclear cells; SIRS = systemic inflammatory response syndrome; LPS = lipopolysaccharide; HS = heat shock; GLN = glutamine; HSP = heat shock protein; mHSP90α = monocyte HSP90α protein expression; ^#^ independent samples Kruskal–Wallis one-way analysis of variance by ranks across groups (significance values have been adjusted by the Bonferroni correction for multiple tests; bold indicates statistical significant difference (*p* < 0.05); * Sepsis vs. Healthy; *** SIRS vs. Healthy); ^^^ related samples Friedman’s two-way analysis of variance by ranks; pairwise comparisons: Wilcoxon signed-ranks test (^^^^ *p* < 0.05 between pairs of no stimulation and stimulation with or without GLN before or after LPS or HS; ^^^^^ between stimulation without GLN and with GLN before or after LPS or HS; ^^^^^^ between stimulation with GLN before and GLN after LPS or HS).

**Table 4:** Comparison analysis of lymphocyte HSP90α expression among different stimulation conditions with or without glutamine pre- or post-treatment in patients with sepsis or trauma, and healthy subjects.

PBMCs from						Healthy Individuals	Patients with Trauma (SIRS)	Patients with Sepsis	
*n* = 40						*n* = 19	*n* = 10	*n* = 11	
Compared Conditions	Time (h)					Lymphocyte HSP90α Expression			
	0	1	2	3	6	Median (IQR)	Median (IQR)	Median (IQR)	^#^*p* Value
Stimulation	None				lHSP90α	46.4 (41.65; 51.9) * ***	60.5 (50.0; 71.15) ***	70.8 (63.8; 83.85) *	**<0.001**
			LPS		lHSP90α	48.2 (42.0; 57.3) *	67.3 (52.2; 73.1)	80.4 (73.9; 152.85) ^^^^^ *	**<0.001**
		GLN	LPS		lHSP90α	46.0 (42.0; 52.55) *	57.3 (48.4; 70.3)	58.0 (51.8; 71.75) ^^^^ *	**0.033**
			LPS	GLN	lHSP90α	48.8 (42.45; 54.3) * ***	64.1 (54.85; 71.6) ***	59.7 (49.5; 68.45) ^^^^ *	**0.013**
						*^^^ p* < 0.422	*^^^ p* = 0.210	*^^^ p* = 0.001	
Stimulation	None				lHSP90α	46.4 (41.65; 51.9) * ***	60.5 (50.0; 71.15) ***	70.8 (63.8; 83.85) *	**<0.001**
			HS		lHSP90α	63.9 (54.75; 73.35) ^^^^ *	66.8 (57.65; 81.6) **	88.4 (76.85; 119.0) * **	**0.002**
		GLN	HS		lHSP90α	57.2 (45.54; 64.15) ^^^^	67.1 (51.75; 82.65)	63.1 (67.7; 84.15) ^^^^^	0.206
			HS	GLN	lHSP90α	60.2 (55.2; 66.3) ^^^^ *	79.9 (65.55; 88.5) ^^^^	73.4 (67.65; 83.95) *	**0.007**
						*^^^ p* < 0.001	*^^^ p* = 0.039	*^^^ p* = 0.024	

Continuous variables are reported as median (interquartile range). Abbreviations: IQR = interquartile range; PBMCs = peripheral blood mononuclear cells; SIRS = Systemic inflammatory response syndrome; LPS = lipopolysaccharide; HS = heat shock; GLN = glutamine; HSP = heat shock protein; lHSP90α = lymphocyte HSP90α protein expression; ^#^ independent samples Kruskal–Wallis one-way analysis of variance by ranks across groups (significance values have been adjusted by the Bonferroni correction for multiple tests; bold indicates statistical significant difference (*p* < 0.05); * Sepsis vs. Healthy controls; ** Sepsis vs. SIRS; *** SIRS vs. Healthy); ^^^ related samples Friedman’s two-way analysis of variance by ranks; pairwise comparisons: Wilcoxon signed-ranks test (^^^^ *p* < 0.05 between pairs of no stimulation and stimulation with or without GLN before or after LPS or HS; ^^^^^ between stimulation without GLN and with GLN before or after LPS or HS.

**Table 5:** Comparison analysis of supernatant cytokine concentrations among different stimulation conditions in patients with sepsis or trauma, and healthy subjects.

PBMCs from					Healthy Individuals	Patients with Trauma (SIRS)	Patients with Sepsis	
*n* = 40					*n* = 19 (15 at 24 h)	*n =* 10 (8 at 24 h)	*n =* 11 (9 at 24 h)	
Compared Conditions	Time (h)				Cytokine Supernatant (pg/mL)			
	0	2	24	48	Median (IQR)	Median (IQR)	Median (IQR)	^#^*p* Value
Stimulation	None		IL-1β		2.00 (0.01; 6.76)	4.87 (0.01; 38.12)	2.00 (1.05; 3.55)	0.664
		LPS	IL-1β		1881 (1382; 2189)	4261 (1576; 7334)	414 (95.75; 2080)	0.058
		HS	IL-1β		0.01 (0.01; 14.46)	5.46 (0.01; 10.33)	2.00 (0.89; 185)	0.775
	None			IL-1β	1.62 (0.01; 6.51)	0.01 (0.01; 8.93)	0.01 (0.01; 2.91)	0.758
		LPS		IL-1β	1338 (1277; 2371)	1863 (363; 3055)	552 (201; 2565)	0.395
		HS		IL-1β	0.01 (0.01; 5.66)	1.96 (0.01; 17.51)	2.00 (0.01; 17.51)	0.734
Stimulation	None		IL-6		2.09 (0.01; 7.32)	6.24 (0.01; 4607)	2.09 (0.01; 7.32)	0.625
		LPS	IL-6		28,154 (21,366; 42,992) ***	120,559 (84,310; 143,653) ***	37549 (8563; 85446)	**0.005**
		HS	IL-6		2.16 (0.01; 25.15)	30.31 (2.92; 164)	2.00 (1.05; 25.30)	0.597
	None			IL-6	6.01 (1.51; 86.79)	0.10 (0.01; 6.24)	3.81 (1.48; 12.23)	0.237
		LPS		IL-6	31,538 (27,992; 40,739)	917,669 (70,412; 135,394)	36,045 (11,817; 77,740)	0.100
		HS		IL-6	4.48 (0.01; 16.82)	119.42 (0.01; 218)	6.62 (3.32; 10.02)	0.525
Stimulation	None		IL-8		3017 (1130; 3256)	3788 (263; 14328)	212 (129; 4969)	0.485
		LPS	IL-8		11,901 (60,841; 180,449) ***	390,558 (255,516; 699,501) ***	211,736 (93,579; 411,038)	**0.010**
		HS	IL-8		1099 (258; 1783)	2127 (812; 4113)	159 (45.53; 3381)	0.294
	None			IL-8	3772 (947; 7463) *	912 (289; 6889)	537 (110; 3690) *	**0.042**
		LPS		IL-8	188,532 (123,770; 285,525) ***	622,075 (318,646; 769,093) ***	114,337 (11,817; 507,894)	**0.015**
		HS		IL-8	512 (289; 2710)	2685 (601; 8462)	235 (66.95; 2736)	0.068
Stimulation	None		IL-10		1.85 (0.01; 4.29)	3.55 (0.01; 30.58)	1.50 (1.08; 2.37)	0.615
		LPS	IL-10		898 (621; 1343) *	713 (414; 1264) **	55.90 (22.06; 120) * **	**0.001**
		HS	IL-10		2.00 (0.01; 4.90)	1.73 (0.01; 5.91)	2.00 (0.66; 2.68)	0.999
	None			IL-10	1.41 (0.01; 4.15)	0.01 (0.01; 7.41)	0.01 (0.01; 1.79)	0.651
		LPS		IL-10	526 (366; 1132) *	162 (115; 698) **	71 (22.14; 148) * **	**<0.001**
		HS		IL-10	0.01 (0.01; 1.46)	0.01 (0.01; 2.12)	1.33 (0.01; 2.36)	0.602
Stimulation	None		TNF-α		1.35 (0.01; 7.91)	4.96 (0.01; 9.88)	0.01 (0.01; 3.83)	0.420
		LPS	TNF-α		922 (781; 1763)	1401 (867; 9263)	1129 (250; 1658)	0.416
		HS	TNF-α		1.58 (0.01; 13.27)	5.36 (0.01; 16.05)	0.01 (0.01; 3.29)	0.736
	None			TNF-α	0.45 (0.01; 4.81)	0.01 (0.01; 1.08)	0.01 (0.01; 0.88)	0.514
		LPS		TNF-α	618 (116; 1636)	115 (74.46; 1742)	170 (76.90; 1154)	0.796
		HS		TNF-α	0.55 (0.01; 3.01)	0.01 (0.01; 4.05)	0.01 (0.01; 1.94)	0.905
Stimulation	None		MCP-1		901 (555; 2179)	986 0.01; 11234)	37.59 (22.52; 110)	0.410
		LPS	MCP-1		811 (0.01; 2919) ***	11,141 (3176; 28,345) ***	4344 (3727; 4891)	**0.029**
		HS	MCP-1		400 (200; 1259)	203 (105; 1556)	49.98 (6.05; 54.9)	0.119
	None			MCP-1	655 (418; 1032) * ***	15.59 (0.01; 253) ***	66.75 (35.16; 194) *	**0.002**
		LPS		MCP-1	5171 (729; 7385) * ***	161,96 (12,400; 32,876) ***	11,883 (2254; 23,562) *	**<0.001**
		HS		MCP-1	1187 (549; 4572)	717 (351; 5784)	53.72 (49.78; 239)	0.199

Continuous variables are reported as median (interquartile range). Abbreviations: IQR = interquartile range; PBMCs = peripheral blood mononuclear cells; SIRS = systemic inflammatory response syndrome; LPS = lipopolysaccharide; HS = heat shock; IL = interleukin; TNF-α = tumor necrosis factor-α; MCP-1 = monocyte chemoattractant protein-1; ^#^ independent samples Kruskal–Wallis one-way analysis of variance by ranks across groups (significance values have been adjusted by the Bonferroni correction for multiple tests; bold indicates statistical significant difference (*p* < 0.05); * Sepsis vs. Healthy; ** Sepsis vs. SIRS; *** SIRS vs. Healthy).

## Data Availability

The datasets generated and analyzed during the current study are not publicly available because the database is very extensive and includes data from other studies complementary to this, but the data are available from the corresponding authors upon reasonable request.

## References

[1] Singer P. (2019). Preserving the Quality of Life: Nutrition in the ICU. Crit. Care.

[2] Vincent J.-L., Jones G., David S., Olariu E., Cadwell K.K. (2019). Frequency and Mortality of Septic Shock in Europe and North America: A Systematic Review and Meta-Analysis. Crit. Care.

[3] Janus P., Kuś P., Vydra N., Toma-Jonik A., Stokowy T., Mrowiec K., Wojtaś B., Gielniewski B., Widłak W. (2022). HSF1 Can Prevent Inflammation Following Heat Shock by Inhibiting the Excessive Activation of the ATF3 and JUN&FOS Genes. Cells.

[4] Walter E.J., Hanna-Jumma S., Carraretto M., Forni L. (2016). The Pathophysiological Basis and Consequences of Fever. Crit. Care.

[5] Drewry A., Mohr N.M. (2022). Temperature Management in the ICU. Crit. Care Med..

[6] Shen Y., Lou Y., Zhu S. (2020). Hyperthermia Is a Predictor of High Mortality in Patients with Sepsis. Crit. Care.

[7] Drewry A.M., Mohr N.M., Ablordeppey E.A., Dalton C.M., Doctor R.J., Fuller B.M., Kollef M.H., Hotchkiss R.S. (2022). Therapeutic Hyperthermia Is Associated with Improved Survival in Afebrile Critically Ill Patients with Sepsis: A Pilot Randomized Trial. Crit. Care Med..

[8] Moura C.S., Lollo P.C.B., Morato P.N., Risso E.M., Amaya-Farfan J. (2017). Modulatory Effects of Arginine, Glutamine and Branched-Chain Amino Acids on Heat Shock Proteins, Immunity and Antioxidant Response in Exercised Rats. Food Funct..

[9] Fitrolaki D.-M., Dimitriou H., Kalmanti M., Briassoulis G. (2013). CD64-Neutrophil Expression and Stress Metabolic Patterns in Early Sepsis and Severe Traumatic Brain Injury in Children. BMC Pediatr..

[10] Fitrolaki M.-D., Dimitriou H., Venihaki M., Katrinaki M., Ilia S., Briassoulis G. (2016). Increased Extracellular Heat Shock Protein 90α in Severe Sepsis and SIRS Associated with Multiple Organ Failure and Related to Acute Inflammatory-Metabolic Stress Response in Children. Medicine.

[11] Vardas K., Apostolou K., Briassouli E., Goukos D., Psarra K., Botoula E., Tsagarakis S., Magira E., Routsi C., Nanas S. (2014). Early Response Roles for Prolactin Cortisol and Circulating and Cellular Levels of Heat Shock Proteins 72 and 90α in Severe Sepsis and SIRS. Biomed Res. Int..

[12] Wischmeyer P.E., Kahana M., Wolfson R., Ren H., Musch M.M., Chang E.B. (2001). Glutamine Induces Heat Shock Protein and Protects against Endotoxin Shock in the Rat. J. Appl. Physiol..

[13] Briassoulis G., Briassouli E., Fitrolaki D.-M., Plati I., Apostolou K., Tavladaki T., Spanaki A.-M. (2014). Heat Shock Protein 72 Expressing Stress in Sepsis: Unbridgeable Gap between Animal and Human Studies--a Hypothetical “Comparative” Study. Biomed Res. Int..

[14] Roth E. (2008). Nonnutritive Effects of Glutamine. J. Nutr..

[15] Cruzat V., Macedo Rogero M., Noel Keane K., Curi R., Newsholme P. (2018). Glutamine: Metabolism and Immune Function, Supplementation and Clinical Translation. Nutrients.

[16] Newsholme P. (2001). Why Is L-Glutamine Metabolism Important to Cells of the Immune System in Health, Postinjury, Surgery or Infection?. J. Nutr..

[17] Cruzat V.F., Pantaleão L.C., Donato J., de Bittencourt P.I.H., Tirapegui J. (2014). Oral Supplementations with Free and Dipeptide Forms of L-Glutamine in Endotoxemic Mice: Effects on Muscle Glutamine-Glutathione Axis and Heat Shock Proteins. J. Nutr. Biochem.

[18] Kao C., Hsu J., Bandi V., Jahoor F. (2013). Alterations in Glutamine Metabolism and Its Conversion to Citrulline in Sepsis. Am. J. Physiol. Endocrinol. Metab..

[19] Rodas P.C., Rooyackers O., Hebert C., Norberg Å., Wernerman J. (2012). Glutamine and Glutathione at ICU Admission in Relation to Outcome. Clin. Sci..

[20] Fan J., Wu J., Wu L.-D., Li G.-P., Xiong M., Chen X., Meng Q.-Y. (2016). Effect of Parenteral Glutamine Supplementation Combined with Enteral Nutrition on Hsp90 Expression and Lymphoid Organ Apoptosis in Severely Burned Rats. Burns.

[21] Li W., Tao S., Wu Q., Wu T., Tao R., Fan J. (2017). Glutamine Reduces Myocardial Cell Apoptosis in a Rat Model of Sepsis by Promoting Expression of Heat Shock Protein 90. J. Surg. Res..

[22] Heyland D., Muscedere J., Wischmeyer P.E., Cook D., Jones G., Albert M., Elke G., Berger M.M., Day A.G. (2013). Canadian Critical Care Trials Group a Randomized Trial of Glutamine and Antioxidants in Critically Ill Patients. N. Engl. J. Med..

[23] van Zanten A.R.H., Sztark F., Kaisers U.X., Zielmann S., Felbinger T.W., Sablotzki A.R., De Waele J.J., Timsit J.-F., Honing M.L.H., Keh D. (2014). High-Protein Enteral Nutrition Enriched with Immune-Modulating Nutrients vs Standard High-Protein Enteral Nutrition and Nosocomial Infections in the ICU: A Randomized Clinical Trial. JAMA.

[24] van Zanten A.R.H., Dhaliwal R., Garrel D., Heyland D.K. (2015). Enteral Glutamine Supplementation in Critically Ill Patients: A Systematic Review and Meta-Analysis. Crit. Care.

[25] Apostolopoulou A., Haidich A.-B., Kofina K., Manzanares W., Bouras E., Tsaousi G., Stoppe C., Dardavessis T.I., Chourdakis M. (2020). Effects of Glutamine Supplementation on Critically Ill Patients: Focus on Efficacy and Safety. An Overview of Systematic Reviews. Nutrition.

[26] Briassoulis G., Filippou O., Hatzi E., Papassotiriou I., Hatzis T. (2005). Early Enteral Administration of Immunonutrition in Critically Ill Children: Results of a Blinded Randomized Controlled Clinical Trial. Nutrition.

[27] Briassoulis G., Filippou O., Kanariou M., Hatzis T. (2005). Comparative Effects of Early Randomized Immune or Non-Immune-Enhancing Enteral Nutrition on Cytokine Production in Children with Septic Shock. Intensive Care Med..

[28] Briassoulis G., Filippou O., Kanariou M., Papassotiriou I., Hatzis T. (2006). Temporal Nutritional and Inflammatory Changes in Children with Severe Head Injury Fed a Regular or an Immune-Enhancing Diet: A Randomized, Controlled Trial. Pediatr. Crit. Care Med..

[29] Jennaro T.S., Viglianti E.M., Ingraham N.E., Jones A.E., Stringer K.A., Puskarich M.A. (2022). Serum Levels of Acylcarnitines and Amino Acids Are Associated with Liberation from Organ Support in Patients with Septic Shock. J. Clin. Med..

[30] Briassoulis P., Ilia S., Briassouli E., Miliaraki M., Briassoulis G. (2021). The Lonely Glutamine Tree in the Middle of the Infinite Critically Ill Forest. Crit. Care.

[31] Briassouli E., Briassoulis G. (2012). Glutamine Randomized Studies in Early Life: The Unsolved Riddle of Experimental and Clinical Studies. Clin. Dev. Immunol..

[32] Briassouli E., Tzanoudaki M., Goukos D., Routsi C., Nanas S., Vardas K., Apostolou K., Kanariou M., Daikos G., Briassoulis G. (2015). Glutamine May Repress the Weak LPS and Enhance the Strong Heat Shock Induction of Monocyte and Lymphocyte HSP72 Proteins but May Not Modulate the HSP72 MRNA in Patients with Sepsis or Trauma. Biomed Res. Int..

[33] Garib R., Garla P., Torrinhas R.S., Moretti A.I.S., Machado M.C.C., Waitzberg D.L. (2016). Effect of Previous High Glutamine Infusion on Inflammatory Mediators and Mortality in an Acute Pancreatitis Model. Mediat. Inflamm..

[34] Fan J., Wu J., Wu L.-D., Cheng B., Tao S.-Y., Wang W., Chen X., Zeng P., Wang Y.-B., Meng Q.-Y. (2018). Effect of Parenteral Glutamine Supplementation Combined with Enteral Nutrition on Hsp90 Expression and Peyer’s Patch Apoptosis in Severely Burned Rats. Nutrition.

[35] Cruzat V.F., Keane K.N., Scheinpflug A.L., Cordeiro R., Soares M.J., Newsholme P. (2015). Alanyl-Glutamine Improves Pancreatic β-Cell Function Following Ex Vivo Inflammatory Challenge. J. Endocrinol..

[36] Briassouli E., Goukos D., Daikos G., Apostolou K., Routsi C., Nanas S., Briassoulis G. (2014). Glutamine Suppresses Hsp72 Not Hsp90α and Is Not Inducing Th1, Th2, or Th17 Cytokine Responses in Human Septic PBMCs. Nutrition.

[37] Shankar-Hari M., Phillips G.S., Levy M.L., Seymour C.W., Liu V.X., Deutschman C.S., Angus D.C., Rubenfeld G.D., Singer M. (2016). Sepsis Definitions Task Force Developing a New Definition and Assessing New Clinical Criteria for Septic Shock: For the Third International Consensus Definitions for Sepsis and Septic Shock (Sepsis-3). JAMA.

[38] Kaukonen K.-M., Bailey M., Pilcher D., Cooper D.J., Bellomo R. (2015). Systemic Inflammatory Response Syndrome Criteria in Defining Severe Sepsis. N. Engl. J. Med..

[39] Basi D.L., Ross K.F., Hodges J.S., Herzberg M.C. (2003). The Modulation of Tissue Factor by Endothelial Cells during Heat Shock. J. Biol. Chem..

[40] Singleton K.D., Beckey V.E., Wischmeyer P.E. (2005). Glutamine prevents activation of NF-kB and Stress Kinase pathways, attenuates inflammatory cytokine release, and prevents Acute Respiratory Distress Syndrome (ARDS) following sepsis. Shock.

[41] Wischmeyer P.E., Riehm J., Singleton K.D., Ren H., Musch M.W., Kahana M., Chang E.B. (2003). Glutamine Attenuates Tumor Necrosis Factor-Alpha Release and Enhances Heat Shock Protein 72 in Human Peripheral Blood Mononuclear Cells. Nutrition.

[42] Newsholme P., Diniz V.L.S., Dodd G.T., Cruzat V. (2022). Glutamine Metabolism and Optimal Immune and CNS Function. Proc. Nutr. Soc..

[43] van Zanten A.R.H., Hofman Z., Heyland D.K. (2015). Consequences of the REDOXS and METAPLUS Trials: The End of an Era of Glutamine and Antioxidant Supplementation for Critically Ill Patients?. JPEN J Parenter Enter. Nutr.

[44] Singer P., Blaser A.R., Berger M.M., Alhazzani W., Calder P.C., Casaer M.P., Hiesmayr M., Mayer K., Montejo J.C., Pichard C. (2019). ESPEN Guideline on Clinical Nutrition in the Intensive Care Unit. Clin. Nutr..

[45] Tume L.N., Valla F.V., Joosten K., Jotterand Chaparro C., Latten L., Marino L.V., Macleod I., Moullet C., Pathan N., Rooze S. (2020). Nutritional Support for Children during Critical Illness: European Society of Pediatric and Neonatal Intensive Care (ESPNIC) Metabolism, Endocrine and Nutrition Section Position Statement and Clinical Recommendations. Intensive Care Med..

[46] Compher C., Bingham A.L., McCall M., Patel J., Rice T.W., Braunschweig C., McKeever L. (2022). Guidelines for the Provision of Nutrition Support Therapy in the Adult Critically Ill Patient: The American Society for Parenteral and Enteral Nutrition. JPEN J. Parenter. Enter. Nutr..

[47] Miliaraki M., Briassoulis P., Ilia S., Polonifi A., Mantzourani M., Briassouli E., Vardas K., Nanas S., Pistiki A., Theodorakopoulou M. (2021). Survivin and Caspases Serum Protein Levels and Survivin Variants MRNA Expression in Sepsis. Sci. Rep..

[48] Gupta S., Lee C.-M., Wang J.-F., Parodo J., Jia S.-H., Hu J., Marshall J.C. (2018). Heat-Shock Protein-90 Prolongs Septic Neutrophil Survival by Protecting c-Src Kinase and Caspase-8 from Proteasomal Degradation. J. Leukoc. Biol..

[49] Yan G., Huang J., Jarbadan N.R., Jiang Y., Cheng H. (2008). Sequestration of NF-KappaB Signaling Complexes in Lipid Rafts Contributes to Repression of NF-KappaB in T Lymphocytes under Hyperthermia Stress. J. Biol. Chem..

[50] Janus P., Stokowy T., Jaksik R., Szoltysek K., Handschuh L., Podkowinski J., Widlak W., Kimmel M., Widlak P. (2015). Cross Talk between Cytokine and Hyperthermia-Induced Pathways: Identification of Different Subsets of NF-ΚB-Dependent Genes Regulated by TNFα and Heat Shock. Mol. Genet. Genom..

[51] Paszek A., Kardyńska M., Bagnall J., Śmieja J., Spiller D.G., Widłak P., Kimmel M., Widlak W., Paszek P. (2020). Heat Shock Response Regulates Stimulus-Specificity and Sensitivity of the pro-Inflammatory NF-ΚB Signalling. Cell Commun. Signal..

[52] Papadopoulos P., Pistiki A., Theodorakopoulou M., Christodoulopoulou T., Damoraki G., Goukos D., Briassouli E., Dimopoulou I., Armaganidis A., Nanas S. (2017). Immunoparalysis: Clinical and Immunological Associations in SIRS and Severe Sepsis Patients. Cytokine.

[53] Kim M., Kim M., Jeong H., Chae J.S., Kim Y.S., Lee J.G., Cho Y., Lee J.H. (2017). Hyporesponsiveness of Natural Killer Cells and Impaired Inflammatory Responses in Critically Ill Patients. BMC Immunol..

[54] West M.A., Koons A. (2008). Endotoxin Tolerance in Sepsis: Concentration-Dependent Augmentation or Inhibition of LPS-Stimulated Macrophage TNF Secretion by LPS Pretreatment. J. Trauma.

[55] Jordan I., Balaguer M., Esteban M.E., Cambra F.J., Felipe A., Hernández L., Alsina L., Molero M., Villaronga M., Esteban E. (2016). Glutamine Effects on Heat Shock Protein 70 and Interleukines 6 and 10: Randomized Trial of Glutamine Supplementation versus Standard Parenteral Nutrition in Critically Ill Children. Clin. Nutr..

[56] Marino L.V., Pathan N., Meyer R., Wright V.J., Habibi P. (2014). The Effect of 2 MMol Glutamine Supplementation on HSP70 and TNF-α Release by LPS Stimulated Blood from Healthy Children. Clin. Nutr..

[57] Marino L.V., Pathan N., Meyer R.W., Wright V.J., Habibi P. (2015). An in Vitro Model to Consider the Effect of 2 MM Glutamine and KNK437 on Endotoxin-Stimulated Release of Heat Shock Protein 70 and Inflammatory Mediators. Nutrition.

[58] Vardas K., Ilia S., Sertedaki A., Charmandari E., Briassouli E., Goukos D., Apostolou K., Psarra K., Botoula E., Tsagarakis S. (2017). Increased Glucocorticoid Receptor Expression in Sepsis Is Related to Heat Shock Proteins, Cytokines, and Cortisol and Is Associated with Increased Mortality. Intensive Care Med. Exp..

[59] Spanaki A.M., Tavladaki T., Dimitriou H., Kozlov A.V., Duvigneau J.C., Meleti E., Weidinger A., Papakonstantinou E., Briassoulis G. (2018). Longitudinal Profiles of Metabolism and Bioenergetics Associated with Innate Immune Hormonal Inflammatory Responses and Amino-Acid Kinetics in Severe Sepsis and Systemic Inflammatory Response Syndrome in Children. JPEN J. Parenter. Enter. Nutr..

[60] Tavladaki T., Spanaki A.M., Dimitriou H., Kondili E., Choulaki C., Georgopoulos D., Briassoulis G. (2017). Similar Metabolic, Innate Immunity, and Adipokine Profiles in Adult and Pediatric Sepsis Versus Systemic Inflammatory Response Syndrome-A Pilot Study. Pediatr. Crit. Care Med..

[61] Huang J., Liu J., Chang G., Wang Y., Ma N., Roy A.C., Shen X. (2021). Glutamine Supplementation Attenuates the Inflammation Caused by LPS-Induced Acute Lung Injury in Mice by Regulating the TLR4/MAPK Signaling Pathway. Inflammation.

[62] Wang H., Dong Y., Cai Y. (2017). Alanyl-glutamine Prophylactically Protects against Lipopolysaccharide-induced Acute Lung Injury by Enhancing the Expression of HSP70. Mol. Med. Rep..

[63] Kaplan J., Nowell M., Chima R., Zingarelli B. (2014). Pioglitazone Reduces Inflammation through Inhibition of NF-ΚB in Polymicrobial Sepsis. Innate Immun..

[64] He L., Zhou X., Wu Z., Feng Y., Liu D., Li T., Yin Y. (2022). Glutamine in Suppression of Lipopolysaccharide-Induced Piglet Intestinal Inflammation: The Crosstalk between AMPK Activation and Mitochondrial Function. Anim. Nutr..

[65] Scharte M., Baba H.A., Van Aken H., Schulzki C., Meyer J., Goeters C., Bone H.G. (2001). Alanyl-Glutamine Dipeptide Does Not Affect Hemodynamics despite a Greater Increase in Myocardial Heat Shock Protein 72 Immunoreactivity in Endotoxemic Sheep. J. Nutr..

[66] Ramnath R.D., Ng S.W., Guglielmotti A., Bhatia M. (2008). Role of MCP-1 in Endotoxemia and Sepsis. Int. Immunopharmacol..

[67] Akhter M.S., Uddin M.A., Kubra K.-T., Barabutis N. (2021). Elucidation of the Molecular Pathways Involved in the Protective Effects of AUY-922 in LPS-Induced Inflammation in Mouse Lungs. Pharmaceuticals.

